# Supplementary material for: Rumor surveillance in support of minimally invasive tissue sampling for diagnosing the cause of child death in low-income countries: A qualitative study
Source: PLoS One. 2021 Jan 28;16(1):e0244552. doi: 10.1371/journal.pone.0244552 (PMC7842994; doi:10.1371/journal.pone.0244552)
Supplement: S1 File — (DOC) [file pone.0244552.s001.doc]

# Interview Guide

**Please record demographic information of every respondent as following:** age, education, occupation, affiliation to the community, religion, years of residing in the community, number of family members, number of < 5 years child in the family, mobile number

1. Please state about the existing practices regarding particularly for early pregnancy loss, stillbirth, neonatal and child (< 5 years of age) death (community perspective). [Please probe for following] Process of informing to the community (who, when and how to inform)

- Religious practices (funeral process)
- Rituals (body preparing, transportation of body, burial)
- Social practices (consoling, social support) done by family members, relatives and community
- Key persons for performing social and other cultural practices in the community
- Any administrative process regarding burial
- Any local efforts to register death and birth
- Role of Community elders in performing these practices
- How important is it to carry out these practices?

1. Can you please state about whether someone is to blame (stigma) for early pregnancy loss or for stillbirth? *[Please probe for following]*

- If so, who is to blame for?
- (If) the case of blaming the mother for child death
- Who is/are involved in blaming activities?
- Pattern of stigma (family and community level)
- Who can be involved in mitigating stigmatization (community level)
- How it can be mitigated

1. What would be the benefits of knowing the cause of a child’s death? Please explain from religious perspective). *[Please probe for following]*
2. Why this information is important or why not (Please brief about the CHAMPS activities) To find out the information on child death, we have to gather some tissue and fluids from the body of child after they die so that we can know what caused the child to die (MITS activities). As a community elder, how would you feel about this being done? *[Please probe for following]*

- Explore any concern from individual perspective
- Explore any concern from the perspective of community, if any concern, how it can be addressed
- (If individual perspective is positive) how can you inform/motivate the community participating in MITS activity

1. Please explain is there any possibility of starting rumors/stigma in the community if tissue and fluids to be collected from deceased child even their parent’s permit? [Please probe for following]

- Pattern of rumor/stigma
- Explore community elders’ role in managing the rumor/stigma
- Suggestions for the CHAMPS to work with the community in mitigating rumors

1. Please mention about appropriate timing regarding the consent for conducting MITS activities

- Timing for consent
- Timing for returning the body

1. Please mention about how consent can be approached for conducting MITS activities? *[Please probe for following]*

- When to approach for consent
- Concerned person/s for decision making and signing for consent
- Any other consideration for consent procedure

1. How would you feel to have photographs of the deceased child?

- Explore any concern on overall photographing of deceased child
- Explore any concern on photographing during observation (for SBS formative study)
- Explore any concern during conducting MITS activities of the deceased child

1. What do you think whether a crest/memento can be provided mentioning the image and/or words regarding the deceased as recognition of participation in this research?

- Explore any concern from perspective of family
- Explore any concern from the perspective of community

1. What do think you about the key persons needed to be involved to inform the community about CHAMPS activities? *[Please probe for following]*

- Religious leader? Why?
- Village chiefs? Why?
- School headmaster? Why?
- Elderly respected person of the community, Why?
- Others, Why?
- Best ways to involve community leaders in CHAMPS activities
- Best ways to educate community people in CHAMPS activities
- Whether their approval is required while conducting MITS despite family members consent in your family, (if yes then) why and how?
