# Supplementary material for: Rumor surveillance in support of minimally invasive tissue sampling for diagnosing the cause of child death in low-income countries: A qualitative study
Source: PLoS One. 2021 Jan 28;16(1):e0244552. doi: 10.1371/journal.pone.0244552 (PMC7842994; doi:10.1371/journal.pone.0244552)
Supplement: S2 File — (DOC) [file pone.0244552.s002.doc]

**ID number of the volunteer:**

|  |  |  |  |  |
| --- | --- | --- | --- | --- |

**Name of the volunteer:………………………………………………………………………………………………………………………...**

**Name of the village where volunteer resides:……………………………………………………………………………………….**

**Put (🗸) mark in front of the appropriate choice**

1. **Which organization runs CHAMPS program?**
2. Icddr,b (Locally known as Cholera Hospital)
3. BRAC
4. ASA
5. Dhaka Medical College Hospital
6. **What the Champs Project deals with?**
7. Prevention of Cholera
8. Prevention of NIPAH virus
9. Infectious diseases
10. Child health and identify the cause of death to prevent infant and child mortality
11. **From whose body did the Champs project collect samples to determine the cause of infant mortality?**
12. Live and healthy child
13. Sick child
14. Deceased child
15. Parents of the child
16. **What instrument has been using to collect the sample from the body of the child to determine the cause of death?**
17. Scissors
18. Knife
19. A special kind of needle
20. Machine
21. **What information a volunteer can inform to the CHAMPS staff?**
22. Death of <5 child and child birth
23. Stillbirth
24. A and B
25. Sickness of child
26. **Truth / False: If we need to take samples from the body of a <5 child who has died at the community or a child delivered stillbirth at home, is it important to know the news of infant death as soon as possible?**
27. True
28. False
29. **If any child died at the community and if the parents of that child agreed to provide the sample to know the cause of death, in which place the sample will be collected?**
30. *Rajbari Sadar* Hospital
31. *Baliakandi Upazila* Health Complex
32. At home
33. At the Union *Porishod* Office (Local administrative office)
34. **If any <5 child died and if you inform it to the CHAMPS hotline number within 4 hours of death, what amount of money will be sent as mobile top-up in your mobile?**
35. 100 Taka
36. 200 Taka
37. 150 Taka
38. 50 Taka
39. **If any <5 child died and if you inform it to the CHAMPS hotline number up to 4 hours to 10 hours of death, what amount of money will be sent as mobile top-up in your mobile?**
40. 100 Taka
41. 200 Taka
42. 150 Taka
43. 50 Taka
44. **If any <5 child died and if you inform it to the CHAMPS hotline number up to 10 hours to 24 hours of death, what amount of money will be sent as mobile top-up in your mobile?**
45. 100 Taka
46. 200 Taka
47. 150 Taka
48. 50 Taka
49. **If any <5 child died and if you inform it to the CHAMPS hotline number after 24 hours of death, what amount of money will be sent as mobile top-up in your mobile?**
50. 100 Taka
51. 200 Taka
52. 150 Taka
53. 50 Taka
54. **Yes / No: if any child dies and if we want to collect sample from the deceased’s body to identify the cause of death, is it necessary to take the permission prior to take the sample?**
55. Yes
56. No
57. **Truth / False: To know the cause of death is the body of the deceased child cut and samples taken?**
58. True
59. False
60. **What can be the role of a community volunteer to prevent any rumor (information deviated from the actual) related to any of the CHAMPS activities?**
61. To inform any of the CHAMPS staff over phone or in person contact
62. To make people inform the actual information from their knowledge what they have learned from this workshop
63. No need to discuss about anything with anyone
64. A and B
65. **True/False: Subject of getting permission from the deceased child to take the sample, a community volunteer can play an important role by informing the death news to the CHAMPS hotline.**
66. True
67. False
